# Pharmacogenetics Update on Biologic Therapy in Psoriasis

**DOI:** 10.3390/medicina56120719

**Published:** 2020-12-20

**Authors:** Ester Muñoz-Aceituno, Luisa Martos-Cabrera, María Carmen Ovejero-Benito, Alejandra Reolid, Francisco Abad-Santos, Esteban Daudén

**Affiliations:** 1 Dermatology Department, Hospital Universitario de la Princesa, 28006 Madrid, Spain; maria.luisa.martoscabrera@gmail.com (L.M.-C.); alereolid@gmail.com (A.R.); estebandauden@gmail.com (E.D.); 2 Clinical Pharmacology Department, Hospital Universitario de La Princesa, Instituto Teófilo Hernando, Faculty of Medicine, Universidad Autónoma de Madrid (UAM), Instituto de Investigación Sanitaria La Princesa (IP), 28006 Madrid, Spain; ovejero.mc@gmail.com (M.C.O.-B.); francisco.abad@salud.madrid.org (F.A.-S.)

**Keywords:** Pharmacogenetics, psoriasis, biologics, anti-IL12/23, anti-IL17

## Abstract

*Background and objectives:* Psoriasis is a chronic immune-mediated skin disease caused by several complex factors, both environmental and genetic, many of which are still not fully understood. Nowadays, several groups of biological drugs are being used for psoriasis treatment. Although these therapies are very effective, they show significant variability in efficacy among individuals. Therefore, there is a need for biomarkers to predict treatment outcomes in order to guide personalized therapeutic decisions. Pharmacogenetics is the study of variations in DNA sequences related to drug response. *Materials and Methods:* In this article, we review pharmacogenetics studies on the treatment of moderate-to-severe psoriasis focusing on anti-interleukin (IL) 12/23 (ustekinumab) and anti-IL17 drugs (secukinumab and ixekizumab), as well as recent studies concerning anti-TNF drugs. *Results:* Several polymorphisms have been studied over the years in reference to anti-TNF drugs; some of the most recent studies included the performance of a genome-wide association study (GWAS) and pharmacogenetics studies focused on the optimization of a treatment regimen. Various polymorphisms in different genes have been related to ustekinumab response; among them, the most commonly studied is the HLA-C*06:02 allele. *Conclusions:* Although not confirmed in some studies, most studies have shown that patients carrying this allele present a significantly higher response rate to ustekinumab. Some polymorphisms have been studied in patients treated with anti-IL17 drugs, mostly related to secukinumab; however, up to now, no association has been found between any of these polymorphisms and response. Nevertheless, further studies involving larger cohorts are needed in order to confirm these results before the implementation of this biomarker in clinical practice.

## 1. Introduction

Psoriasis is a chronic immune-mediated skin disease that is known to affect around 2-3% of the world population [1]. In its etiopathology there is involvement of T-helper cell type 1 (Th1) and type 17 (Th17) inflammation, a high rate of epidermal proliferation, abnormal keratinocyte differentiation and blood vessel dilatation accompanied by angiogenesis [2]. There are multiple types of clinical presentation of psoriasis depending on the appearance of the lesions, and the most common form is plaque psoriasis (affecting approximately 90% of psoriasis patients). Other less common forms include guttate, pustular, and erythrodermic psoriasis. The Psoriasis Area and Severity Index (PASI) and body surface affected (BSA) scores allow clinicians to classify the severity of psoriasis. The absolute PASI, or the percentage of PASI improvement over the baseline value (e.g., PASI75, a 75% reduction in PASI), can be useful for evaluating the effectiveness of a treatment. Psoriasis has chronic and inflammatory components that not only affect the skin, but can play a relevant role in the appearance of a multitude of comorbidities such as hypertension, dyslipidemia, fatty liver, psychosocial conditions or inflammatory bowel disease, being responsible for a high burden of comorbidity worldwide [1,3]. 

Psoriasis is caused by several complex factors, both environmental and genetic, many of which are still not fully understood [4,5]. Nowadays, genetic studies are useful tools to identify potential new targets for psoriasis treatment. With advances in single nucleotide polymorphism (SNP) microarray technology and large-scale genome databases, genome-wide association studies (GWAS) have been conducted to study multiple traits and diseases [5]. Molecular genetics studies have identified more than 80 genomic loci involved in about 30% of psoriasis heritability, which show variation associated with risk of disease [6] Like several other multifactorial autoimmune disorders, psoriasis manifests strong associations with HLA, mostly with HLA-C, in particular with the HLA-C*06:02 allele, which was the first risk allele identified for psoriasis, presenting certain differences amongst different races [5,7].

Currently, there is no definite cure for psoriasis, but there are multiple treatments available to relieve the symptoms and signs of the disease. Moderate-to-severe psoriasis patients not effectively responding to topical therapies (corticosteroids, vitamin D and analogues, among others) or phototherapy are usually treated with systemic therapies. Conventional systemic drugs include methotrexate, ciclosporin A and acitretin, but a high percentage of patients do not respond to this kind of therapy [8]. Increased knowledge of psoriasis pathogenesis has accelerated the development of more selective and effective anti-inflammatory agents, called biologics, which are manufactured antibodies or fusion proteins that block proinflammatory cytokines or their receptors [9]. Currently, several groups of biological drugs are available for the treatment of psoriasis: anti-TNF (such as adalimumab, etanercept or infliximab), anti-IL12/23 (ustekinumab), anti-IL17 (secukinumab, ixekizumab), anti-IL17 receptor (brodalumab) and anti-IL23 (guselkumab, risankizumab and tildrakizumab). However, the response to these agents is heterogeneous. It is hypothesized that at least 50–70% of this inter-individual variation could be explained by a polymorphic genetic background [10]. 

Pharmacogenetics is the science that studies the genetic variations that are involved in the different responses to pharmacological therapies for any disease. Its main objective is to comprehend the reason why certain polymorphisms mean that a person has greater or lesser possibility of suffering adverse effects with the same drug, making it possible to use the safest drug for each patient from the first moment, thus also increasing the compliance. Another of its main objectives is to understand why the presence of certain polymorphisms makes the metabolism of a drug faster or slower in different patients and therefore its effectiveness is decreased or increased. It has been defined by the US FDA as ‘‘the study of variations in DNA sequence as related to drug response’’ [11,12,13,14].

Pharmacogenetics research on psoriasis treatments is an expanding field. The main studies are focused on SNPs of genes known to encode proteins and drug transporters/receptors involved in the pharmacokinetics or pharmacodynamics of certain drugs. SNPs are substitutions of one nucleotide occurring in at least 1% of the population [12,15,16].

Pharmacogenomics applies pharmacogenetics to the whole genome to observe the impact of genetic variations on drug response in patients; it is defined as the study of variations in the characteristics of DNA and RNA associated to drug response [11]. Pharmacogenomic studies are mostly based on GWAS, which test simultaneously numerous SNPs (over 800,000) throughout the entire genome [17,18].

Various studies have been published about the pharmacogenetics of biological drugs, especially of anti-TNF [12,19,20,21]. The aim of this review is to present an update of the main pharmacogenetic findings in psoriasis focusing on the latest data on anti-TNF drugs, as well as a comprehensive review on pharmacogenetics of anti-IL12/23 and anti-IL17. To our knowledge, there are currently no studies regarding anti-IL23 drugs.

## 2. Materials and Methods

A search for scientific evidence was carried out using MEDLINE and the Cochrane library, with a search from January 1st of 1999 to June 1st of 2020. The search strategy used in MEDLINE was (psoriasis) and (pharmacogenetics or pharmacogenomics or polymorphisms or snp) and (biologics or treatment or tnf or anti IL17 or anti IL12 / 23 or anti IL23 or adalimumab or etanercept or ustekinumab or secukinumab or ixekizumab or brodalumab or guselkumab or tildrakizumab or risankizumab), obtaining 962 references. 

The search strategy carried out in the Cochrane library was (psoriasis) and (pharmacogenetics or pharmacogenomics) and (biologics or anti tnf or anti IL17 or anti IL12 / 23 or adalimumab or etanercept or ustekinumab or secukinumab or ixekizumab or brochumab or guselkumab or tildizumab risankizumab), obtaining 2 results. 

In the first phase, a title reading was performed. If the article could not be excluded by reading the title, the abstract was read and those articles that were not clearly related to the review were excluded. After reviewing the title and abstract, 857 articles were excluded. Therefore, 105 articles were finally evaluated.

## 3. Anti-TNF Drugs

Anti-TNF drugs available for psoriasis treatment (etanercept, infliximab and adalimumab) are generally well-tolerated and effective drugs. Nevertheless, up to 50% of the patients do not show enough clinical improvement with these drugs [22]. In addition, although not frequently, they can be the source of severe adverse events such as paradoxical psoriasiform reactions [23]. Multiple studies on the pharmacogenetics of response to anti-TNF drugs published along the years have been thoroughly reviewed in several recent publications. [12,19,20,21] Since fewer articles have been published recently concerning this topic [24,25,26,27], we focused our review on drugs that have been studied in more detail in recent years. 

Most pharmacogenetics studies on anti-TNF drugs were designed following a candidate-gene approach, i.e., analyzing a reduced number of genes previously associated with psoriasis or biological drug response [12,24,28,29,30,31,32,33,34,35,36,37,38,39,40,41,42,43,44]. On the contrary, only two studies were conducted using GWAS, thus following a pharmacogenomics approach [27,45]. Nevertheless, GWAS studies have failed to identify SNPs significantly associated with biological drug response in psoriasis. These results may be partially explained by a reduced sample size.

Currently, most patients failing to achieve a good response using anti-TNF drugs are switched to anti-IL12/23, anti-IL17 or anti-IL23. Nevertheless, when using lifelong biological treatments, it is necessary to reduce the probability of side effects. Moreover, biological drugs are expensive, which translates into high healthcare costs worldwide [46,47,48,49]. However, off label optimization (dose reduction or increase in the interval of drug administration) of biological drug dosage represents a therapeutic alternative for patients who exhibit an excellent response in clinical practice [46,47,48,49,50]. In a previous publication by our group, we found an association of SNPs located on *LMO4, IL28RA, CYLD, IL12B, TNFAIP3* and *VEGFA* genes with overall and successful dose reduction for biological drugs (*N* = 120). Additionally, we found an association between successful adalimumab dose reduction and polymorphisms in *IL28RA, SDC4*, *TLR10, TRAF3IP2* and *MICA-A9* (*N* = 61). These SNPs could help to anticipate which patients could be subjected to biological drug optimization, thus reducing the risk of adverse events and increasing cost-effectiveness. Nevertheless, these findings should be confirmed in further studies before their implementation in clinical practice [25].

## 4. Anti-IL12/23 Drugs (Ustekinumab)

Ustekinumab is a human monoclonal antibody that binds to the p40 subunit shared by IL12 and IL23 and is an effective treatment for psoriasis. Most of the pharmacogenetics studies on this drug have focused on the HLA-C*06: 02 allele (see Table 1). HLA-C*06:02 status is associated with different presentations of psoriasis. HLA-C*06:02 positive patients experience earlier onset, higher incidence of the Koebner phenomenon, and augmented likelihood of worsening of the lesions following streptococcal throat infection [7,51]. Moreover, each copy of the HLA-C*06:02 allele carried causes a five-fold increase in the risk of psoriasis [52]. Currently, HLA-C*06:02 is the best-studied biomarker related to ustekinumab response, showing results supporting the suggestion that its presence predisposes to a more rapid and prolonged response [51,53,54,55,56,57,58]. However, new larger studies are needed to consider its use in routine clinical practice. The functional role of HLA-C in the pathogenesis of psoriasis is unclear; the major role of HLA-C has been assumed to be related to antigen presentation to CD8+ T-cells. The migration of this type of cell towards the epidermis seems to be a requirement for the onset of psoriatic lesions [59]. One possible explanation for the close relationship of this biomarker with treatment response could be that HLA-C*06:02 patients suffer from an endotype of psoriasis which is highly dependent on IL12/23 signaling that would make them more sensitive to the blockade of this pathway [53]. Another hypothesis postulates that this psoriasis subtype may be triggered by autoantigens presented in the context of HLA-C*06:02 to CD8+ T-cells, which are abundant in psoriasis epidermis and are highly dependent on IL23 [60].

One study involving 51 patients treated with ustekinumab found a striking difference in the rate of ustekinumab responders between HLA-C*06:02 positive and HLA-C*06:02 negative patients with an increased response to ustekinumab in patients carrying the HLA-C*06:02 allele. [53] This effect of HLA-C*06:02 was confirmed by the same authors with two larger cohorts of 134 [54] and later of 255 Caucasian patients treated with ustekinumab. [55] The latter study showed that patients carrying the HLA-C*06:02 allele responded faster to treatment at week 4; PASI50 was achieved at week 4 in 71.7% and 35.2% of HLA-C*06:02 positive and HLA-C*06:02 negative patients, respectively. A significant difference was also observed between these two groups of patients in the proportion of PASI90 achievement after 4 weeks. Moreover, 51.2% of HLA-C*06:02 positive patients reached PASI 90 at week 12 and 65.2% at week 52, while only 25.0% and 42.5% of HLA-C*06:02 negative patients reached PASI 90 at weeks 12 and 52, respectively. The authors concluded that HLA-C*06:02 positivity predisposed patients to a faster and longer lasting response to ustekinumab. Another study in 64 patients who had been treated with ustekinumab for up to 1 year established that HLA-C*06:02 positive patients were better responders to ustekinumab [57]. In addition, a study carried out in Portuguese population analyzed HLA-C polymorphisms and their association with PASI 75 at weeks 4, 12, 24 and 52 in 116 patients treated with ustekinumab. HLA-C*06:02 positive patients responded better at week 12 and 24, but not at week 52. Therefore, the authors suggested that this polymorphism could be a marker of early response to ustekinumab [58]. HLA-C*06:02 was also studied in a large cohort of 1326 patients, analyzing PASI 90 at months 3, 6 and 12, comparing ustekinumab (487 patients) and adalimumab (839 patients) treated patients. This study established that HLA-C*06:02-negative patients were more likely to respond to adalimumab than to ustekinumab treatment (most strongly at 6 months), mainly if they also presented psoriatic arthritis. The authors concluded that along with the presence of psoriatic arthritis, studying HLA-C*06:02 could be of great help in seeking the best first-line treatment for psoriasis patients also supporting the hypothesis that HLA-C*06:02 positive patients may present a different endotype of psoriasis [51].

However, contrary to what has been reported by other authors, a Spanish study in 69 ustekinumab patients did not find a significant association between the response to ustekinumab and SNPs in HLA-C [61]. Similarly, a large North American study of 601 psoriasis patients from phase III clinical trials of ustekinumab in moderate-to-severe psoriasis also studied the HLA C*06:02 allele observing a modestly higher proportion of HLA-C*06:02 positive patients achieving PASI75/90 responses at weeks 12 and 24. The authors concluded that the modest difference in response between HLA-C*06:02 positive and HLAC*06:02 negative patients does not provide a clear rationale for using HLA-C*06:02 genotyping for guiding the choice of therapy [56]. Moreover, a study in a Chinese cohort of 29 patients treated with ustekinumab found that positivity for HLA-C*06:02 was detrimental to ustekinumab response, although this is the only publication reporting this negative association, and it should be taken into account that the cohort was small and the results did not reach statistical significance [62]. Nevertheless, the same authors in a later study analyzed HLA-C polymorphisms, finding that HLA-C*06:02-positive patients had a better response, in accordance with previous studies; for example, at week 28, a significantly higher percentage of HLA-C*06:02 positive patients maintained PASI90 response compared with HLA-C*06:02 negative patients (63% vs. 26%, *p* = 0.035) [63].

Besides HLA-C, the association with response to ustekinumab has been also studied for polymorphisms in other genes. For example, a study on a small cohort of 22 Greek psoriasis patients treated with ustekinumab genotyped 38 variants that had been significantly associated with psoriasis in genome-wide studies, finding that only two SNPs (rs151823 and rs26653 [*ERAP1*]) showed an association with a good response to ustekinumab. They determined that other genes such as *TRAF3IP2, TNFAIP3*, or *HLA-A* were associated with a response to anti-TNF but not to ustekinumab [35]. A study in 234 patients, in which 66 ustekinumab episodes were registered, analyzed the copy number variation in *LCE3B* and *3C* genes, as well as eight SNPs in *HLA-C, TNFAIP3, CD84, IL23R, TRAF3IP2, ERAP1, IL12b* and *IFIH1*. Heterozygous patients (CT) for rs3213094 (*IL12b*) showed a statistically significant better response to ustekinumab than the reference group (CC), whereas homozygous patients (GG) for rs610604 (*TNFAIP3*) showed worse response to ustekinumab than the reference group (TT) [64]. Evaluation of ten SNPs in genes involved in Th17 pathways, such as *IL23R, IL23A*, and *IL12B*, showed no significant association between any of these SNPs and PASI75 or PASI90 response to ustekinumab [56]. A study conducted in a Danish population including 230 patients treated with ustekinumab genotyped a total of 44 genes and 62 SNPs. Out of them, only four SNPs in three genes (rs1143623, rs1143627 [*IL1B*], rs8177374 [*TIRAP*] and rs5744174 [*TLR5*]) were significantly associated with response to ustekinumab treatment (PASI75 for responders versus <PASI50 for non-responders) after 3 months of treatment. The authors concluded that genetic variants in genes involved in regulating the cytokines more strongly associated with the pathophysiology of psoriasis were related to therapy response. In addition, genetic variants that were associated with higher levels of IL-1β were related to worse response to treatment and those associated with a greater expression of INF-γ made the patient more predisposed to respond better to ustekinumab [24]. Another pharmacogenetic study in a Spanish population found an association between rs763780 (*IL17F*) and response to ustekinumab (*N* = 70) at 3 and 6 months, whereas rs2275913 and rs10484879 (*IL17A*) did not show any influence on the response [65]. A posterior study by our group (*N*=69) found an association between SNPs in *C17orf51, ZNF816A, C9orf72, STAT4, CHUK,* and *SLC22A4* genes and a good response to ustekinumab, whereas SNPs in *TNFRSF1A, HTR2A, NFKBIA, ADAM33,* and *IL13* genes were associated with a poor response to this drug [61]. Patients lacking the AA genotype for the rs3212227 (*IL12B*) SNP but carrying the GG genotype for the rs6887695 (*IL12B*) SNP were found to respond better to ustekinumab. However, no relation to response was found for SNPS in *TNFAIP3, IL12B, IL23R* or for *IL6 LCE3B/C* deletion [57]. Moreover, no relationship with a response to ustekinumab was found for rs1120926 (*IL23R*) and rs6887695 (*IL12B*) [58], or for rs610604 (*TNFAIP3*) polymorphism and *LCE3B/3C* gene deletions. [53] Regarding optimization, in a previous study by our group, we observed a correlation between successful ustekinumab dose reduction and polymorphisms in *LMO4, NLRP3* and *LELP1* (*N* = 55) [25].

**Table 1:** Pharmacogenetics studies on ustekinumab showing significant associations between polymorphisms and response to treatment. SNP: single nucleotide polymorphism, HLA: human leukocyte antigens, PASI: Psoriasis Area and Severity Index, IL: interleukin * Therapeutic success increases in HLA-C*06:02 positive patients.

Reference	Genes	Protein Function	Allele/SNP	Response	Number of Patients	Country	Weeks after Initiation of Treatment	Outcome Measure
Talamonti et al, 2013 [53]	*HLA-C*	It belongs to the HLA class I heavy chain group. Relevant role in the immune system as it helps to present endoplasmic reticulum peptides.	HLA-C*06:02 positive	Better	51	Italy	4, 12, 28 and 40	PASI 75. PASI 90
Chiu et al, 2014 [63]	*HLA-C*	It belongs to the HLA class I heavy chain group. Relevant role in the immune system as it helps to present endoplasmic reticulum peptides.	HLA-C*06:02 positive	Better	66	China	16 and 28	PASI50, PASI75, PASI90
Prieto-Pérez et al, 2015 [65]	*IL-17F*	This cytokine is expressed by activated T cells, and is involved, among others, in IL-23 and IL-17 Family Signaling Pathways in the immune response.	rs763780 (TC genotype)	Worse	70	Spain	12 and 24	PASI75
Masouri et al, 2016 [35]	*ERAP1*	Aminopeptidase involved in trimming precursors of HLA class I-binding peptides to allow their presentation by MHC class I molecules	rs151823 (CC genotype)	Better	22	Greece	24	PASI 50, PASI75
			rs26653 (GG genotype)	Better				
Li et al, 2016 [56]	*HLA-C*	It belongs to the HLA class I heavy chain group. Relevant role in the immune system as it helps to present endoplasmic reticulum peptides.	HLA-C*06:02 positive	Better	601	The United States of America	4, 12, 24 and 28	PASI50, PASI75, PASI 90.PASI100
Galluzo et al, 2016 [57]	*HLA-C*	It belongs to the HLA class I heavy chain group. Relevant role in the immune system as it helps to present endoplasmic reticulum peptides.	HLA-C*06:02 positive	Better	64	Italy	4, 12, 28, 40 and 52	PASI 75
Talamonti et al, 2016 [54]	*HLA-C*	It belongs to the HLA class I heavy chain group. Relevant role in the immune system as it helps to present endoplasmic reticulum peptides.	HLA-C*06:02 positive	Better	134	Italy	4, 12, 28, 52, 76, 104, 156	PASI75
Talamonti et al, 2017 [55]	*HLA-C*	It belongs to the HLA class I heavy chain group. Relevant role in the immune system as it helps to present endoplasmic reticulum peptides.	HLA-C*06:02 positive	Better	255	Italy	4, 12, 28, 40 and 52	PASI 50, PASI75, PASI90
Raposo et al, 2017 [58]	*HLA-C*	It belongs to the HLA class I heavy chain group. Relevant role in the immune system as it helps to present endoplasmic reticulum peptides.	HLA-C*06:02 positive	Better	116	Portugal	4, 12, 24 and 52	PASI75
Reek et al, 2017 [64]	*IL12b*	Subunit of interleukin 12, expressed by activated macrophages and essential inducer of Th1 cell development.	rs3213094 (CT genotype)	Better	Number of patients not stated. Sixty-six episodes on ustekinumab	The Netherlands	6, 12, and every three months thereafter	PASI75, ΔPASI
	*TNFAIP3*	Zinc finger protein and ubiquitin-editing enzyme.	rs610604 (GG genotype)	Worse				
Prieto-Pérez et al, 2017 [61]	*TNFRSF1A*	Member of the TNF receptor superfamily of proteins. Plays a role in cell survival, apoptosis, and inflammation.	rs191190 (TT genotype)	Worse	69	Spain	16	PASI75
	*HTR2A*	Serotonin receptor. Mutations in this gene are associated with susceptibility to schizophrenia and obsessive-compulsive disorder.	rs6311 (TT genotype)	Worse				
	*NFKBIA*	Member of the NF-kappa-B inhibitor family.	rs2145623 (CC genotype)	Worse				
	*ADAM33*	Member of the ADAM (a disintegrin and metalloprotease domain) family.	rs2787094 (CC genotype)	Worse				
	*IL13*	Immunoregulatory cytokine produced primarily by activated Th2 cells.	rs848 (TT genotype)	Worse				
	*CHUK*	Serine kinase that plays an essential role in the NF-kappa-B signaling pathway.	rs11591741 (GC genotype)	Better				
	*C17orf51*	RNA Gene associated with psoriasis	rs1975974 (AG genotype)	Better				
	*ZNF816A*	Transcriptional regulation	rs9304742 (CT genotype)	Better				
	*STAT4*	Mediating responses to IL12 in lymphocytes, and regulating the differentiation of T helper cells.	rs7574865 (GT genotype)	Better				
	*SLC22A4*	Polyspecific organic cation transporter critical for elimination of drugs and environmental toxins.	rs1050152 (CT genotype)	Better				
	*C9orf72*	Important role in the regulation of endosomal trafficking. Interacts with Rab proteins, which are involved in autophagy and endocytic transport.	rs774359 (CT genotype)	Better				
Loft et al, 2018 [24]	*IL1B*	Important mediator of the inflammatory response.	rs1143623 (G/C genotype)	Better	230	Denmark	12, 24	PASI50, PASI75
			rs1143627 (T/C genotype)	Better				
	*TIRAP*	TIR adaptor protein involved in the toll-like receptor signaling pathway in the immune system.	rs8177374 (C/T genotype)	Better				
	*TLR5*	Member of the TLR family, which plays a fundamental role in pathogen recognition and activation of the innate immune response.	rs5744174 (T/C genotype)	Better				
	*Il12B*	Subunit of interleukin 12. This cytokine is expressed by activated macrophages and is an essential inducer of Th1 cells development.	rs6887695 (GG genotype)	Better*				
			rs3212227 (absence of AA genotype)	Better*				
Dand et al, 2019 [51]	*HLA- C*	It belongs to the HLA class I heavy chain group. Relevant role in the immune system as it helps to present endoplasmic reticulum peptides.	HLA-C*06:02 positive	Better.	487	The United Kingdom	12, 24, 48	PASI 75, PASI90, PASI100

## 5. Anti-IL17 Drugs

Secukinumab and ixekizumab are fully human monoclonal antibodies that target IL17A. Ixekizumab also binds to the heterodimer form of the protein (IL17A/F). These drugs have shown that they achieve a very rapid initial response that is fortunately maintained over time with a very good safety profile [66,67]. There are very few studies evaluating the pharmacogenetics of anti-IL17 and anti-IL17 receptor drugs. All of them focus on secukinumab, while only one of them also includes patients on ixekizumab treatment (see Table 2).

The influence of HLA-C*06:02 status on the efficacy and safety of secukinumab was studied in a phase III clinical trial of patients with moderate-to-severe psoriasis (SUPREME study). The study analyzed a cohort of 434 patients and found no differences in PASI90 after 16 weeks of treatment between HLA-C*06:02 positive and negative patients: PASI90 was achieved in 80.4% of HLA-C*06:02 positive and 79.7% of HLA-C*06:02 negative patients. At week 24, PASI100 responses were similar in both cohorts (HLA-C*06:02 positive, 62.0%; HLA-C*06:02 negative, 61.0%). In addition, no differences were found either in the mean absolute PASI at week 16, or in the median time to reach PASI90. Moreover, no statistical differences in safety were observed between both groups. Taken together, these results indicate that HLA-C*06:02 is not a good candidate marker for secukinumab therapy, as this treatment is highly effective regardless of the HLA-C*06:02 status [68]. An extension phase of the SUPREME study confirmed these results, establishing that secukinumab was equally effective in both HLA-C*06:02-positive and HLA-C*06:02-negative patients with PASI response rates comparable between both groups up to week 72 [69].

Similar results were found in another work with a small cohort of 18 patients undergoing treatment with secukinumab, in which no relationship was found between HLA-C*06:02 and response after three months of treatment. However, using published data to estimate the sample size required for predicting success of ustekinumab treatment at 16 weeks with HLA-C*06:02, the authors established that a minimum of 216 patients would be needed to confirm a difference in response (α = 0.05, power= 90%) [70].

A multicenter study recruited 134 patients from four different European hospitals treated with secukinumab (118 patients) and ixekizumab (16 patients). Using a sequencing approach, the protein coding and untranslated regions of the *IL17A* gene were studied in these patients. This analysis showed that the protein-coding region of the *IL17A* gene was invariable amongst those 134 patients. Five variants found in the non-coding regions (rs3748067, rs2275913, rs3819025, rs7747909 and rs8193037) had been known or suspected to have a functional effect on *IL17A* gene expression. However, they did not show any association with the response to these drugs after 12 weeks of treatment [71].

**Table 2:** Pharmacogenetics studies on anti-IL17 drugs. HLA: human leukocyte antigens, PASI: Psoriasis Area and Severity Index, IL: interleukin.

Reference	Drug	Genes Studied	Response	Number of Patients	Country	Weeks after Initiation of Treatment	Outcome Measure
Costanzo et al, 2018 [68]	Secukinumab	*HLA-C*	HLA-C*06:02 had no relation to response	434	Italy	16 and 24	PASI50, PASI75, PASI90, PASI100
Anzengruber et al, 2018 [70]	Secukinumab	*HLA-C*	HLA-C*06:02 had no relation to response	18	Switzerland	12	PASI50, PASI75, PASI90
Papini et al, 2019 [69]	Secukinumab	*HLA-C*	HLA-C*06:02 had no relation to response	434	Italy	1, 2, 3, 4, 8, 12, 16, 20, 24, 36, 48, 60 and 72	PASI75, PASI90, PASI100
Vugt et al, 2020 [71]	Secukinumab and ixekizumab	*IL-17*	No relation to response	134	The Netherlands, Belgium, Italy, Estonia	12 and 24	PASI75, PASI90

## 6. Conclusions and Future Directions

The association with ustekinumab response has been studied for several polymorphisms. Nevertheless, their usefulness as response biomarkers may vary among different populations depending on polymorphism prevalence. Therefore, in the future, potential biomarkers for ustekinumab found in small studies should be validated in wide international cohorts. With regard to anti-IL-17 drugs, new studies are needed in order to search for predictive markers of response. Similarly, pharmacogenetics studies should be performed for newer biologics (brodalumab, guselkumab, tildrakizumab, and risankizumab) and small molecules (apremilast). Furthermore, it would be interesting for future studies on these drugs to focus not solely on the search for polymorphisms associated with efficacy, but also on finding predictors for toxicity or dose optimization able to help us to identify which patients will maintain a good response despite dose reduction and which patients will be less likely to experience adverse effects. Clearly, identifying those patients more likely to respond to a certain treatment and less likely to suffer adverse effects would be a very significant advance in the treatment of patients with psoriasis. This would reduce the cost and morbidity associated with these therapies. In order to achieve this, we need studies with large cohorts of patients, whose results should be posteriorly replicated in independent cohorts. On many occasions, large replication studies carried out in the past have been unable to confirm potential markers of treatment response. This may be due to the fact that a wide variety of genetic and environmental factors, rather than a single main factor, play a relevant role in this pathology. Personalized medicine is a goal that must be achieved through large cohorts of well-characterized patients with long-term exposure to the drug.
